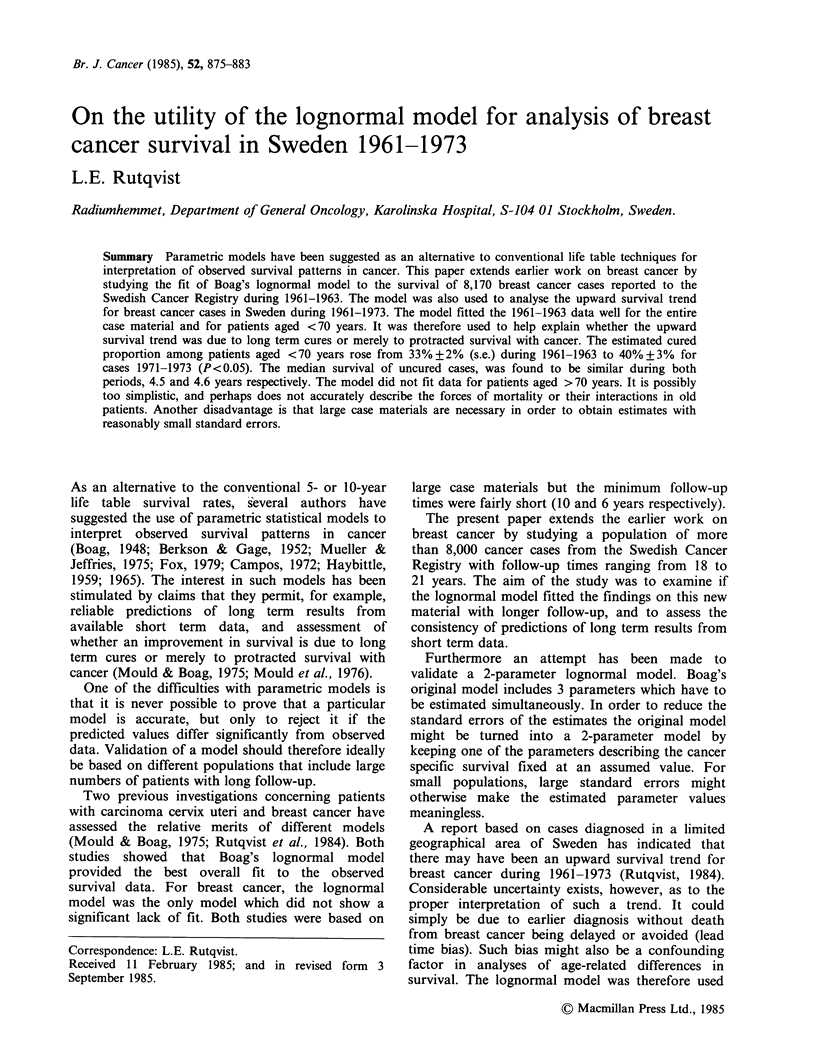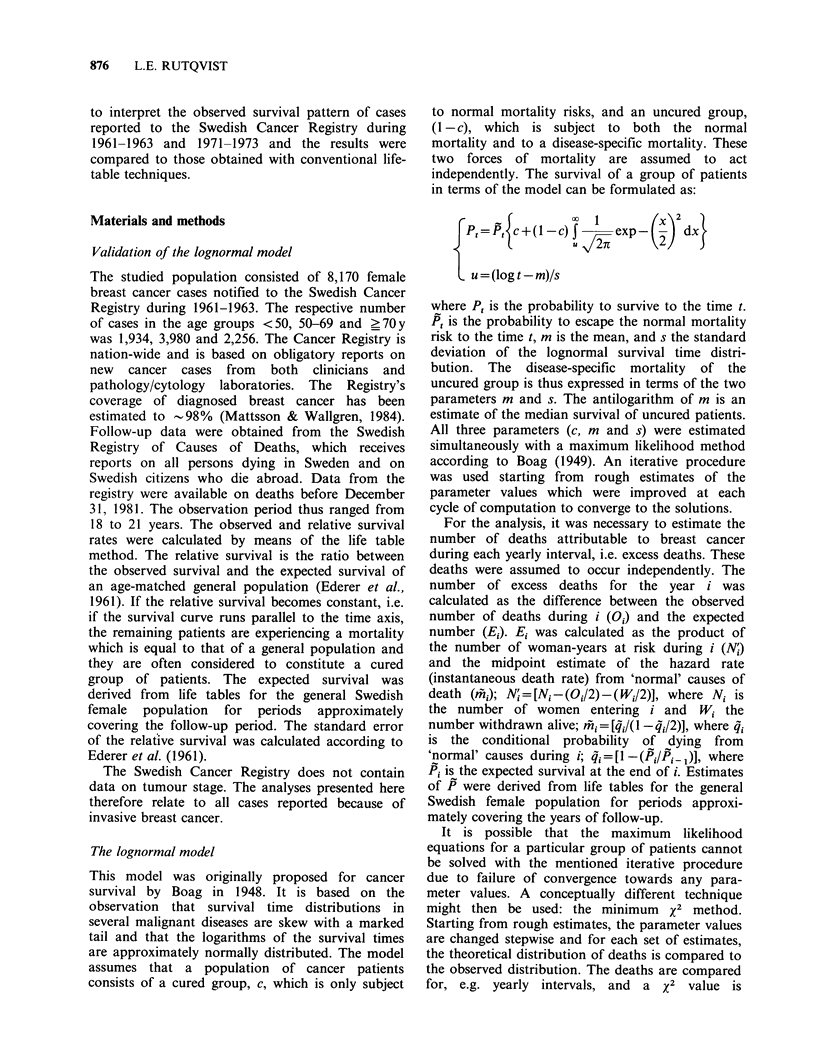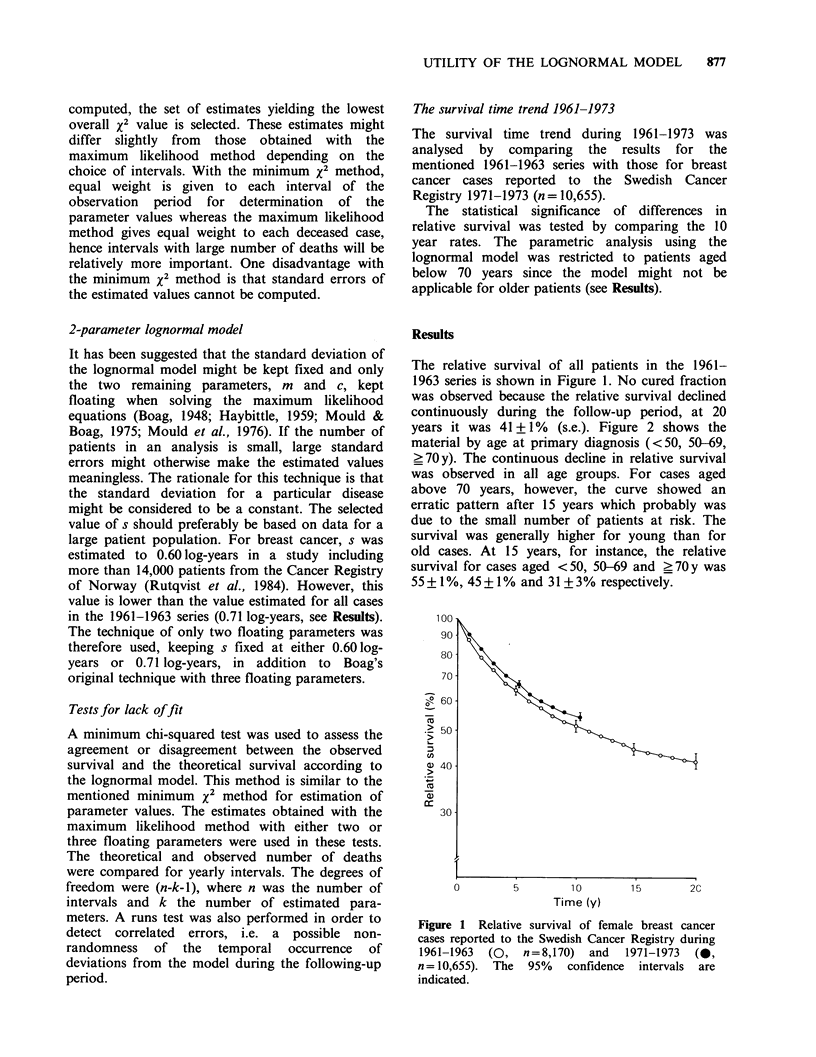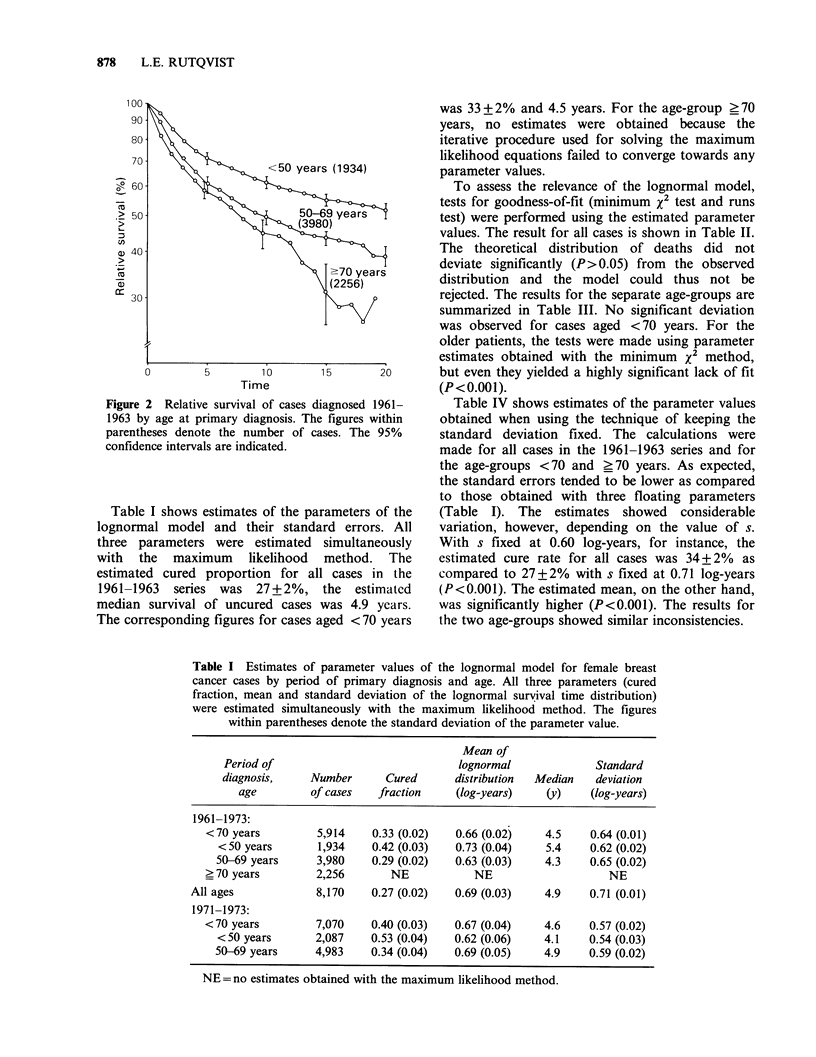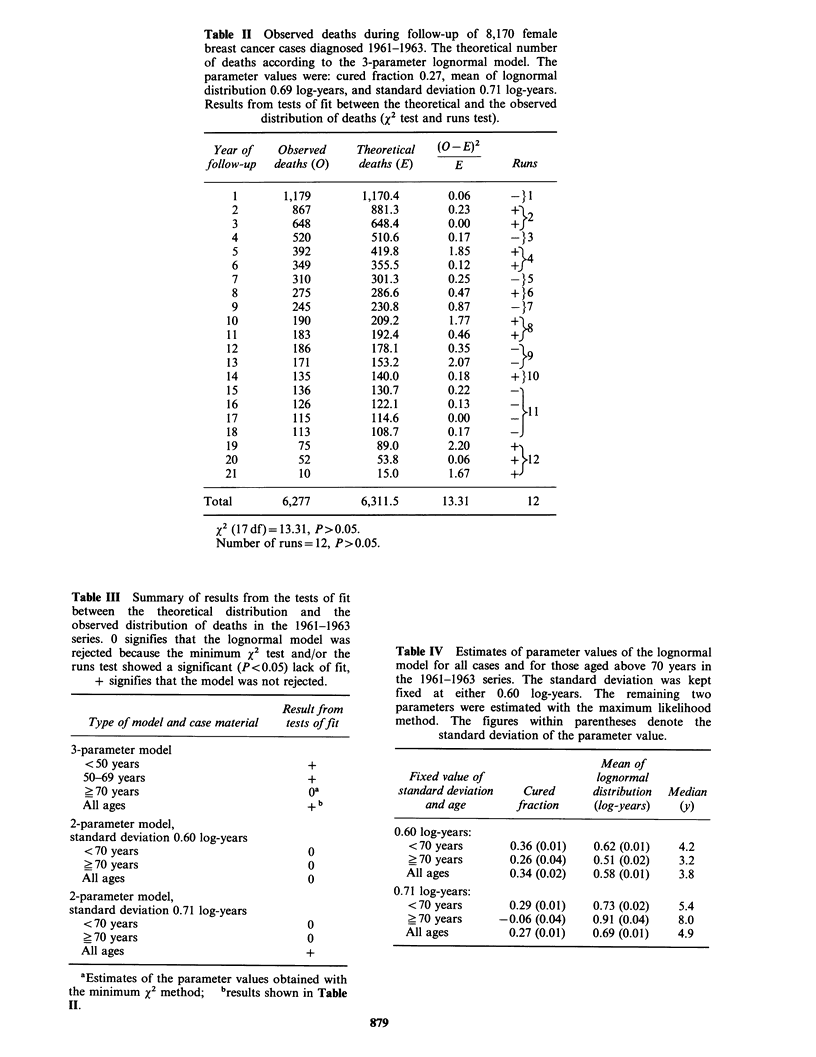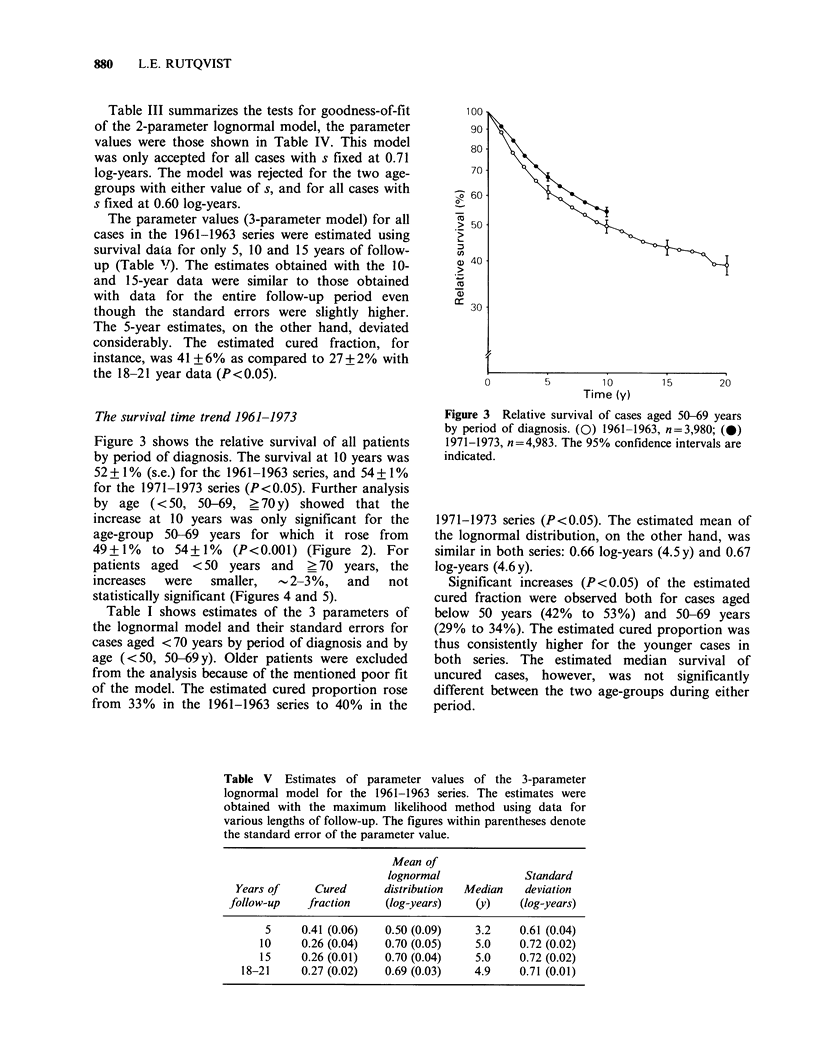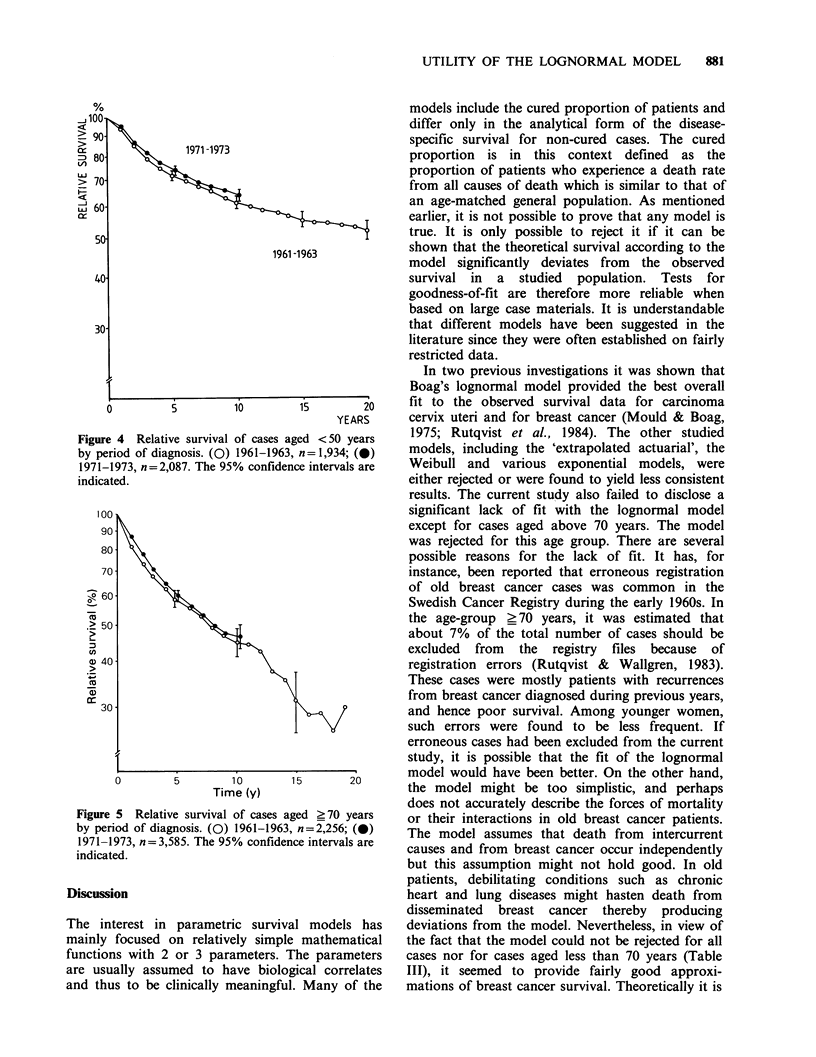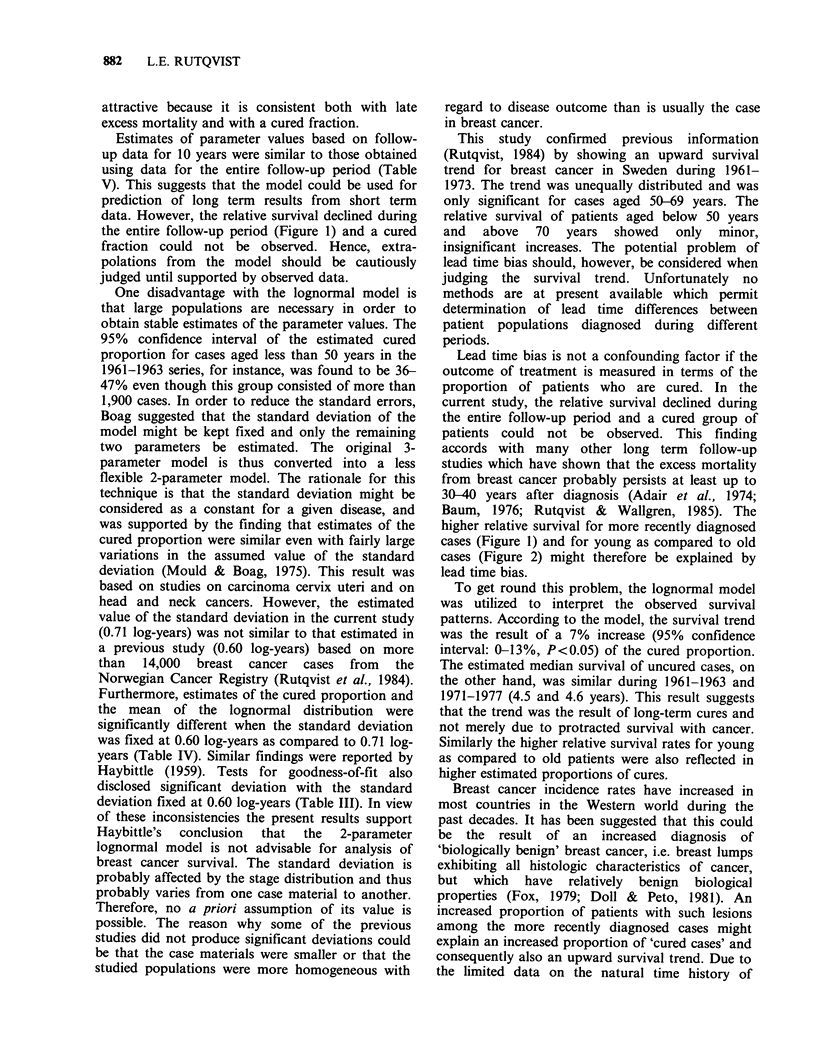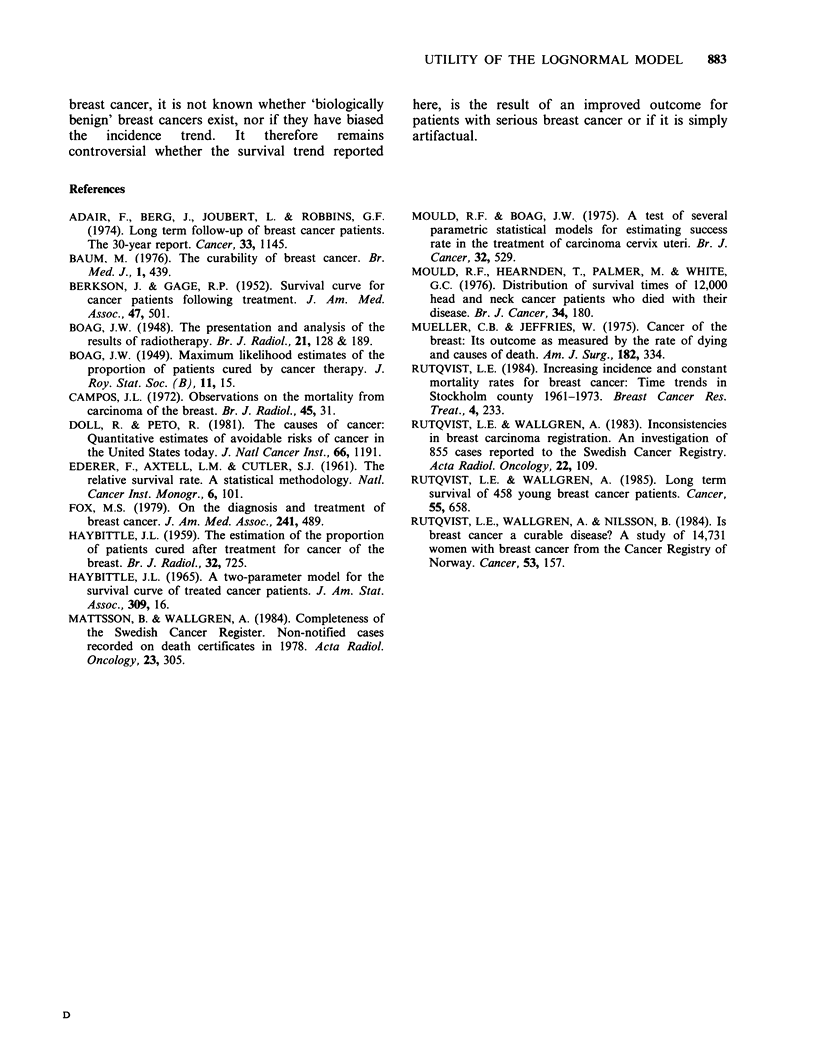# On the utility of the lognormal model for analysis of breast cancer survival in Sweden 1961-1973.

**DOI:** 10.1038/bjc.1985.272

**Published:** 1985-12

**Authors:** L. E. Rutqvist

## Abstract

Parametric models have been suggested as an alternative to conventional life table techniques for interpretation of observed survival patterns in cancer. This paper extends earlier work on breast cancer by studying the fit of Boag's lognormal model to the survival of 8,170 breast cancer cases reported to the Swedish Cancer Registry during 1961-1963. The model was also used to analyse the upward survival trend for breast cancer cases in Sweden during 1961-1973. The model fitted the 1961-1963 data well for the entire case material and for patients aged less than 70 years. It was therefore used to help explain whether the upward survival trend was due to long term cures or merely to protracted survival with cancer. The estimated cured proportion among patients aged less than 70 years rose from 33% +/- 2% (s.e.) during 1961-1963 to 40% +/- 3% for cases 1971-1973 (P less than 0.05). The median survival of uncured cases, was found to be similar during both periods, 4.5 and 4.6 years respectively. The model did not fit data for patients aged greater than 70 years. It is possibly too simplistic, and perhaps does not accurately describe the forces of mortality or their interactions in old patients. Another disadvantage is that large case materials are necessary in order to obtain estimates with reasonably small standard errors.


*Br. J. Cancer* (1985), **52**, 875–883

### On the utility of the lognormal model for analysis of breast cancer survival in Sweden 1961–1973

L.E. Rutqvist

*Radiumhemmet, Department of General Oncology, Karolinska Hospital, S-104 01 Stockholm, Sweden.*

**Summary** Parametric models have been suggested as an alternative to conventional life table techniques for interpretation of observed survival patterns in cancer. This paper extends earlier work on breast cancer by studying the fit of Boag's lognormal model to the survival of 8,170 breast cancer cases reported to the Swedish Cancer Registry during 1961–1963. The model was also used to analyse the upward survival trend for breast cancer cases in Sweden during 1961–1973. The model fitted the 1961–1963 data well for the entire case material and for patients aged $<70$ years. It was therefore used to help explain whether the upward survival trend was due to long term cures or merely to protracted survival with cancer. The estimated cured proportion among patients aged $<70$ years rose from $33\% \pm 2\%$ (s.e.) during 1961–1963 to $40\% \pm 3\%$ for cases 1971–1973 ($P<0.05$). The median survival of uncured cases, was found to be similar during both periods, 4.5 and 4.6 years respectively. The model did not fit data for patients aged $>70$ years. It is possibly too simplistic, and perhaps does not accurately describe the forces of mortality or their interactions in old patients. Another disadvantage is that large case materials are necessary in order to obtain estimates with reasonably small standard errors.

As an alternative to the conventional 5- or 10-year life table survival rates, several authors have suggested the use of parametric statistical models to interpret observed survival patterns in cancer (Boag, 1948; Berkson & Gage, 1952; Mueller & Jeffries, 1975; Fox, 1979; Campos, 1972; Haybittle, 1959; 1965). The interest in such models has been stimulated by claims that they permit, for example, reliable predictions of long term results from available short term data, and assessment of whether an improvement in survival is due to long term cures or merely to protracted survival with cancer (Mould & Boag, 1975; Mould *et al.*, 1976).

One of the difficulties with parametric models is that it is never possible to prove that a particular model is accurate, but only to reject it if the predicted values differ significantly from observed data. Validation of a model should therefore ideally be based on different populations that include large numbers of patients with long follow-up.

Two previous investigations concerning patients with carcinoma cervix uteri and breast cancer have assessed the relative merits of different models (Mould & Boag, 1975; Rutqvist *et al.*, 1984). Both studies showed that Boag's lognormal model provided the best overall fit to the observed survival data. For breast cancer, the lognormal model was the only model which did not show a significant lack of fit. Both studies were based on large case materials but the minimum follow-up times were fairly short (10 and 6 years respectively).

The present paper extends the earlier work on breast cancer by studying a population of more than 8,000 cancer cases from the Swedish Cancer Registry with follow-up times ranging from 18 to 21 years. The aim of the study was to examine if the lognormal model fitted the findings on this new material with longer follow-up, and to assess the consistency of predictions of long term results from short term data.

Furthermore an attempt has been made to validate a 2-parameter lognormal model. Boag's original model includes 3 parameters which have to be estimated simultaneously. In order to reduce the standard errors of the estimates the original model might be turned into a 2-parameter model by keeping one of the parameters describing the cancer specific survival fixed at an assumed value. For small populations, large standard errors might otherwise make the estimated parameter values meaningless.

A report based on cases diagnosed in a limited geographical area of Sweden has indicated that there may have been an upward survival trend for breast cancer during 1961–1973 (Rutqvist, 1984). Considerable uncertainty exists, however, as to the proper interpretation of such a trend. It could simply be due to earlier diagnosis without death from breast cancer being delayed or avoided (lead time bias). Such bias might also be a confounding factor in analyses of age-related differences in survival. The lognormal model was therefore used

Correspondence: L.E. Rutqvist.
Received 11 February 1985; and in revised form 3 September 1985.

© Macmillan Press Ltd., 1985

to interpret the observed survival pattern of cases reported to the Swedish Cancer Registry during 1961–1963 and 1971–1973 and the results were compared to those obtained with conventional life-table techniques.

### Materials and methods

### *Validation of the lognormal model*

The studied population consisted of 8,170 female breast cancer cases notified to the Swedish Cancer Registry during 1961–1963. The respective number of cases in the age groups <50, 50–69 and ≧70 y was 1,934, 3,980 and 2,256. The Cancer Registry is nation-wide and is based on obligatory reports on new cancer cases from both clinicians and pathology/cytology laboratories. The Registry's coverage of diagnosed breast cancer has been estimated to ~98% (Mattsson & Wallgren, 1984). Follow-up data were obtained from the Swedish Registry of Causes of Deaths, which receives reports on all persons dying in Sweden and on Swedish citizens who die abroad. Data from the registry were available on deaths before December 31, 1981. The observation period thus ranged from 18 to 21 years. The observed and relative survival rates were calculated by means of the life table method. The relative survival is the ratio between the observed survival and the expected survival of an age-matched general population (Ederer *et al.*, 1961). If the relative survival becomes constant, i.e. if the survival curve runs parallel to the time axis, the remaining patients are experiencing a mortality which is equal to that of a general population and they are often considered to constitute a cured group of patients. The expected survival was derived from life tables for the general Swedish female population for periods approximately covering the follow-up period. The standard error of the relative survival was calculated according to Ederer *et al.* (1961).

The Swedish Cancer Registry does not contain data on tumour stage. The analyses presented here therefore relate to all cases reported because of invasive breast cancer.

### *The lognormal model*

This model was originally proposed for cancer survival by Boag in 1948. It is based on the observation that survival time distributions in several malignant diseases are skew with a marked tail and that the logarithms of the survival times are approximately normally distributed. The model assumes that a population of cancer patients consists of a cured group, $c$, which is only subject to normal mortality risks, and an uncured group, $(1-c)$, which is subject to both the normal mortality and to a disease-specific mortality. These two forces of mortality are assumed to act independently. The survival of a group of patients in terms of the model can be formulated as:

$$\begin{cases} P_t = \tilde{P}_t\left\{c + (1-c)\int_u^{\infty} \frac{1}{\sqrt{2\pi}} \exp -\left(\frac{x}{2}\right)^2 dx\right\} \\ u = (\log t - m)/s \end{cases}$$

where $P_t$ is the probability to survive to the time $t$. $\tilde{P}_t$ is the probability to escape the normal mortality risk to the time $t$, $m$ is the mean, and $s$ the standard deviation of the lognormal survival time distribution. The disease-specific mortality of the uncured group is thus expressed in terms of the two parameters $m$ and $s$. The antilogarithm of $m$ is an estimate of the median survival of uncured patients. All three parameters ($c$, $m$ and $s$) were estimated simultaneously with a maximum likelihood method according to Boag (1949). An iterative procedure was used starting from rough estimates of the parameter values which were improved at each cycle of computation to converge to the solutions.

For the analysis, it was necessary to estimate the number of deaths attributable to breast cancer during each yearly interval, i.e. excess deaths. These deaths were assumed to occur independently. The number of excess deaths for the year $i$ was calculated as the difference between the observed number of deaths during $i$ ($O_i$) and the expected number ($E_i$). $E_i$ was calculated as the product of the number of woman-years at risk during $i$ ($N_i'$) and the midpoint estimate of the hazard rate (instantaneous death rate) from 'normal' causes of death ($\tilde{m}_i$); $N_i' = [N_i - (O_i/2) - (W_i/2)]$, where $N_i$ is the number of women entering $i$ and $W_i$ the number withdrawn alive; $\tilde{m}_i = [\tilde{q}_i/(1-\tilde{q}_i/2)]$, where $\tilde{q}_i$ is the conditional probability of dying from 'normal' causes during $i$; $\tilde{q}_i = [1-(\tilde{P}_i/\tilde{P}_{i-1})]$, where $\tilde{P}_i$ is the expected survival at the end of $i$. Estimates of $\tilde{P}$ were derived from life tables for the general Swedish female population for periods approximately covering the years of follow-up.

It is possible that the maximum likelihood equations for a particular group of patients cannot be solved with the mentioned iterative procedure due to failure of convergence towards any parameter values. A conceptually different technique might then be used: the minimum $\chi^2$ method. Starting from rough estimates, the parameter values are changed stepwise and for each set of estimates, the theoretical distribution of deaths is compared to the observed distribution. The deaths are compared for, e.g. yearly intervals, and a $\chi^2$ value is computed, the set of estimates yielding the lowest overall $\chi^2$ value is selected. These estimates might differ slightly from those obtained with the maximum likelihood method depending on the choice of intervals. With the minimum $\chi^2$ method, equal weight is given to each interval of the observation period for determination of the parameter values whereas the maximum likelihood method gives equal weight to each deceased case, hence intervals with large number of deaths will be relatively more important. One disadvantage with the minimum $\chi^2$ method is that standard errors of the estimated values cannot be computed.

*2-parameter lognormal model*

It has been suggested that the standard deviation of the lognormal model might be kept fixed and only the two remaining parameters, *m* and *c*, kept floating when solving the maximum likelihood equations (Boag, 1948; Haybittle, 1959; Mould & Boag, 1975; Mould *et al.*, 1976). If the number of patients in an analysis is small, large standard errors might otherwise make the estimated values meaningless. The rationale for this technique is that the standard deviation for a particular disease might be considered to be a constant. The selected value of *s* should preferably be based on data for a large patient population. For breast cancer, *s* was estimated to 0.60 log-years in a study including more than 14,000 patients from the Cancer Registry of Norway (Rutqvist *et al.*, 1984). However, this value is lower than the value estimated for all cases in the 1961–1963 series (0.71 log-years, see **Results**). The technique of only two floating parameters was therefore used, keeping *s* fixed at either 0.60 log-years or 0.71 log-years, in addition to Boag's original technique with three floating parameters.

*Tests for lack of fit*

A minimum chi-squared test was used to assess the agreement or disagreement between the observed survival and the theoretical survival according to the lognormal model. This method is similar to the mentioned minimum $\chi^2$ method for estimation of parameter values. The estimates obtained with the maximum likelihood method with either two or three floating parameters were used in these tests. The theoretical and observed number of deaths were compared for yearly intervals. The degrees of freedom were (*n*-*k*-1), where *n* was the number of intervals and *k* the number of estimated parameters. A runs test was also performed in order to detect correlated errors, i.e. a possible non-randomness of the temporal occurrence of deviations from the model during the following-up period.

*The survival time trend 1961–1973*

The survival time trend during 1961–1973 was analysed by comparing the results for the mentioned 1961–1963 series with those for breast cancer cases reported to the Swedish Cancer Registry 1971–1973 ($n = 10,655$).

The statistical significance of differences in relative survival was tested by comparing the 10 year rates. The parametric analysis using the lognormal model was restricted to patients aged below 70 years since the model might not be applicable for older patients (see **Results**).

### Results

The relative survival of all patients in the 1961–1963 series is shown in Figure 1. No cured fraction was observed because the relative survival declined continuously during the follow-up period, at 20 years it was $41 \pm 1\%$ (s.e.). Figure 2 shows the material by age at primary diagnosis (<50, 50–69, ≧70 y). The continuous decline in relative survival was observed in all age groups. For cases aged above 70 years, however, the curve showed an erratic pattern after 15 years which probably was due to the small number of patients at risk. The survival was generally higher for young than for old cases. At 15 years, for instance, the relative survival for cases aged <50, 50–69 and ≧70 y was $55 \pm 1\%$, $45 \pm 1\%$ and $31 \pm 3\%$ respectively.

**Figure 1** Relative survival of female breast cancer cases reported to the Swedish Cancer Registry during 1961–1963 (○, $n = 8,170$) and 1971–1973 (●, $n = 10,655$). The 95% confidence intervals are indicated.

**Figure 2** Relative survival of cases diagnosed 1961–1963 by age at primary diagnosis. The figures within parentheses denote the number of cases. The 95% confidence intervals are indicated.

Table I shows estimates of the parameters of the lognormal model and their standard errors. All three parameters were estimated simultaneously with the maximum likelihood method. The estimated cured proportion for all cases in the 1961–1963 series was 27±2%, the estimated median survival of uncured cases was 4.9 years. The corresponding figures for cases aged <70 years was 33±2% and 4.5 years. For the age-group ≧70 years, no estimates were obtained because the iterative procedure used for solving the maximum likelihood equations failed to converge towards any parameter values.

To assess the relevance of the lognormal model, tests for goodness-of-fit (minimum $\chi^2$ test and runs test) were performed using the estimated parameter values. The result for all cases is shown in Table II. The theoretical distribution of deaths did not deviate significantly ($P>0.05$) from the observed distribution and the model could thus not be rejected. The results for the separate age-groups are summarized in Table III. No significant deviation was observed for cases aged <70 years. For the older patients, the tests were made using parameter estimates obtained with the minimum $\chi^2$ method, but even they yielded a highly significant lack of fit ($P<0.001$).

Table IV shows estimates of the parameter values obtained when using the technique of keeping the standard deviation fixed. The calculations were made for all cases in the 1961–1963 series and for the age-groups <70 and ≧70 years. As expected, the standard errors tended to be lower as compared to those obtained with three floating parameters (Table I). The estimates showed considerable variation, however, depending on the value of $s$. With $s$ fixed at 0.60 log-years, for instance, the estimated cure rate for all cases was 34±2% as compared to 27±2% with $s$ fixed at 0.71 log-years ($P<0.001$). The estimated mean, on the other hand, was significantly higher ($P<0.001$). The results for the two age-groups showed similar inconsistencies.

**Table I** Estimates of parameter values of the lognormal model for female breast cancer cases by period of primary diagnosis and age. All three parameters (cured fraction, mean and standard deviation of the lognormal survival time distribution) were estimated simultaneously with the maximum likelihood method. The figures within parentheses denote the standard deviation of the parameter value.

| *Period of diagnosis, age* | *Number of cases* | *Cured fraction* | *Mean of lognormal distribution (log-years)* | *Median (y)* | *Standard deviation (log-years)* |
|---|---|---|---|---|---|
| 1961–1973: | | | | | |
| <70 years | 5,914 | 0.33 (0.02) | 0.66 (0.02) | 4.5 | 0.64 (0.01) |
| <50 years | 1,934 | 0.42 (0.03) | 0.73 (0.04) | 5.4 | 0.62 (0.02) |
| 50–69 years | 3,980 | 0.29 (0.02) | 0.63 (0.03) | 4.3 | 0.65 (0.02) |
| ≧70 years | 2,256 | NE | NE | | NE |
| All ages | 8,170 | 0.27 (0.02) | 0.69 (0.03) | 4.9 | 0.71 (0.01) |
| 1971–1973: | | | | | |
| <70 years | 7,070 | 0.40 (0.03) | 0.67 (0.04) | 4.6 | 0.57 (0.02) |
| <50 years | 2,087 | 0.53 (0.04) | 0.62 (0.06) | 4.1 | 0.54 (0.03) |
| 50–69 years | 4,983 | 0.34 (0.04) | 0.69 (0.05) | 4.9 | 0.59 (0.02) |

NE = no estimates obtained with the maximum likelihood method.

**Table II** Observed deaths during follow-up of 8,170 female breast cancer cases diagnosed 1961–1963. The theoretical number of deaths according to the 3-parameter lognormal model. The parameter values were: cured fraction 0.27, mean of lognormal distribution 0.69 log-years, and standard deviation 0.71 log-years. Results from tests of fit between the theoretical and the observed distribution of deaths ($\chi^2$ test and runs test).

| *Year of follow-up* | *Observed deaths (O)* | *Theoretical deaths (E)* | $\frac{(O-E)^2}{E}$ | *Runs* |
|---|---|---|---|---|
| 1 | 1,179 | 1,170.4 | 0.06 | − } 1 |
| 2 | 867 | 881.3 | 0.23 | + } 2 |
| 3 | 648 | 648.4 | 0.00 | + |
| 4 | 520 | 510.6 | 0.17 | − } 3 |
| 5 | 392 | 419.8 | 1.85 | + } 4 |
| 6 | 349 | 355.5 | 0.12 | + |
| 7 | 310 | 301.3 | 0.25 | − } 5 |
| 8 | 275 | 286.6 | 0.47 | + } 6 |
| 9 | 245 | 230.8 | 0.87 | − } 7 |
| 10 | 190 | 209.2 | 1.77 | + } 8 |
| 11 | 183 | 192.4 | 0.46 | + |
| 12 | 186 | 178.1 | 0.35 | − } 9 |
| 13 | 171 | 153.2 | 2.07 | − |
| 14 | 135 | 140.0 | 0.18 | + } 10 |
| 15 | 136 | 130.7 | 0.22 | − } 11 |
| 16 | 126 | 122.1 | 0.13 | − |
| 17 | 115 | 114.6 | 0.00 | − |
| 18 | 113 | 108.7 | 0.17 | − |
| 19 | 75 | 89.0 | 2.20 | + } 12 |
| 20 | 52 | 53.8 | 0.06 | + |
| 21 | 10 | 15.0 | 1.67 | + |
| Total | 6,277 | 6,311.5 | 13.31 | 12 |

$\chi^2$ (17 df) = 13.31, $P > 0.05$.
Number of runs = 12, $P > 0.05$.

**Table III** Summary of results from the tests of fit between the theoretical distribution and the observed distribution of deaths in the 1961–1963 series. 0 signifies that the lognormal model was rejected because the minimum $\chi^2$ test and/or the runs test showed a significant ($P < 0.05$) lack of fit, + signifies that the model was not rejected.

| *Type of model and case material* | *Result from tests of fit* |
|---|---|
| 3-parameter model | |
| < 50 years | + |
| 50–69 years | + |
| ≧ 70 years | 0[a] |
| All ages | +[b] |
| 2-parameter model, standard deviation 0.60 log-years | |
| < 70 years | 0 |
| ≧ 70 years | 0 |
| All ages | 0 |
| 2-parameter model, standard deviation 0.71 log-years | |
| < 70 years | 0 |
| ≧ 70 years | 0 |
| All ages | + |

[a]Estimates of the parameter values obtained with the minimum $\chi^2$ method; [b]results shown in **Table II**.

**Table IV** Estimates of parameter values of the lognormal model for all cases and for those aged above 70 years in the 1961–1963 series. The standard deviation was kept fixed at either 0.60 log-years. The remaining two parameters were estimated with the maximum likelihood method. The figures within parentheses denote the standard deviation of the parameter value.

| *Fixed value of standard deviation and age* | *Cured fraction* | *Mean of lognormal distribution (log-years)* | *Median (y)* |
|---|---|---|---|
| 0.60 log-years: | | | |
| < 70 years | 0.36 (0.01) | 0.62 (0.01) | 4.2 |
| ≧ 70 years | 0.26 (0.04) | 0.51 (0.02) | 3.2 |
| All ages | 0.34 (0.02) | 0.58 (0.01) | 3.8 |
| 0.71 log-years: | | | |
| < 70 years | 0.29 (0.01) | 0.73 (0.02) | 5.4 |
| ≧ 70 years | −0.06 (0.04) | 0.91 (0.04) | 8.0 |
| All ages | 0.27 (0.01) | 0.69 (0.01) | 4.9 |

Table III summarizes the tests for goodness-of-fit of the 2-parameter lognormal model, the parameter values were those shown in Table IV. This model was only accepted for all cases with $s$ fixed at 0.71 log-years. The model was rejected for the two age-groups with either value of $s$, and for all cases with $s$ fixed at 0.60 log-years.

The parameter values (3-parameter model) for all cases in the 1961–1963 series were estimated using survival data for only 5, 10 and 15 years of follow-up (Table V). The estimates obtained with the 10- and 15-year data were similar to those obtained with data for the entire follow-up period even though the standard errors were slightly higher. The 5-year estimates, on the other hand, deviated considerably. The estimated cured fraction, for instance, was 41±6% as compared to 27±2% with the 18–21 year data ($P<0.05$).

*The survival time trend 1961–1973*

Figure 3 shows the relative survival of all patients by period of diagnosis. The survival at 10 years was 52±1% (s.e.) for the 1961–1963 series, and 54±1% for the 1971–1973 series ($P<0.05$). Further analysis by age (<50, 50–69, ≧70 y) showed that the increase at 10 years was only significant for the age-group 50–69 years for which it rose from 49±1% to 54±1% ($P<0.001$) (Figure 2). For patients aged <50 years and ≧70 years, the increases were smaller, ~2–3%, and not statistically significant (Figures 4 and 5).

Table I shows estimates of the 3 parameters of the lognormal model and their standard errors for cases aged <70 years by period of diagnosis and by age (<50, 50–69 y). Older patients were excluded from the analysis because of the mentioned poor fit of the model. The estimated cured proportion rose from 33% in the 1961–1963 series to 40% in the 1971–1973 series ($P<0.05$). The estimated mean of the lognormal distribution, on the other hand, was similar in both series: 0.66 log-years (4.5 y) and 0.67 log-years (4.6 y).

Significant increases ($P<0.05$) of the estimated cured fraction were observed both for cases aged below 50 years (42% to 53%) and 50–69 years (29% to 34%). The estimated cured proportion was thus consistently higher for the younger cases in both series. The estimated median survival of uncured cases, however, was not significantly different between the two age-groups during either period.

**Figure 3** Relative survival of cases aged 50–69 years by period of diagnosis. (○) 1961–1963, $n$=3,980; (●) 1971–1973, $n$=4,983. The 95% confidence intervals are indicated.

**Table V** Estimates of parameter values of the 3-parameter lognormal model for the 1961–1963 series. The estimates were obtained with the maximum likelihood method using data for various lengths of follow-up. The figures within parentheses denote the standard error of the parameter value.

| *Years of follow-up* | *Cured fraction* | *Mean of lognormal distribution (log-years)* | *Median (y)* | *Standard deviation (log-years)* |
|---|---|---|---|---|
| 5 | 0.41 (0.06) | 0.50 (0.09) | 3.2 | 0.61 (0.04) |
| 10 | 0.26 (0.04) | 0.70 (0.05) | 5.0 | 0.72 (0.02) |
| 15 | 0.26 (0.01) | 0.70 (0.04) | 5.0 | 0.72 (0.02) |
| 18–21 | 0.27 (0.02) | 0.69 (0.03) | 4.9 | 0.71 (0.01) |

**Figure 4** Relative survival of cases aged <50 years by period of diagnosis. (○) 1961–1963, $n = 1,934$; (●) 1971–1973, $n = 2,087$. The 95% confidence intervals are indicated.

**Figure 5** Relative survival of cases aged ≧70 years by period of diagnosis. (○) 1961–1963, $n = 2,256$; (●) 1971–1973, $n = 3,585$. The 95% confidence intervals are indicated.

### Discussion

The interest in parametric survival models has mainly focused on relatively simple mathematical functions with 2 or 3 parameters. The parameters are usually assumed to have biological correlates and thus to be clinically meaningful. Many of the models include the cured proportion of patients and differ only in the analytical form of the disease-specific survival for non-cured cases. The cured proportion is in this context defined as the proportion of patients who experience a death rate from all causes of death which is similar to that of an age-matched general population. As mentioned earlier, it is not possible to prove that any model is true. It is only possible to reject it if it can be shown that the theoretical survival according to the model significantly deviates from the observed survival in a studied population. Tests for goodness-of-fit are therefore more reliable when based on large case materials. It is understandable that different models have been suggested in the literature since they were often established on fairly restricted data.

In two previous investigations it was shown that Boag's lognormal model provided the best overall fit to the observed survival data for carcinoma cervix uteri and for breast cancer (Mould & Boag, 1975; Rutqvist *et al.,* 1984). The other studied models, including the 'extrapolated actuarial', the Weibull and various exponential models, were either rejected or were found to yield less consistent results. The current study also failed to disclose a significant lack of fit with the lognormal model except for cases aged above 70 years. The model was rejected for this age group. There are several possible reasons for the lack of fit. It has, for instance, been reported that erroneous registration of old breast cancer cases was common in the Swedish Cancer Registry during the early 1960s. In the age-group ≧70 years, it was estimated that about 7% of the total number of cases should be excluded from the registry files because of registration errors (Rutqvist & Wallgren, 1983). These cases were mostly patients with recurrences from breast cancer diagnosed during previous years, and hence poor survival. Among younger women, such errors were found to be less frequent. If erroneous cases had been excluded from the current study, it is possible that the fit of the lognormal model would have been better. On the other hand, the model might be too simplistic, and perhaps does not accurately describe the forces of mortality or their interactions in old breast cancer patients. The model assumes that death from intercurrent causes and from breast cancer occur independently but this assumption might not hold good. In old patients, debilitating conditions such as chronic heart and lung diseases might hasten death from disseminated breast cancer thereby producing deviations from the model. Nevertheless, in view of the fact that the model could not be rejected for all cases nor for cases aged less than 70 years (Table III), it seemed to provide fairly good approximations of breast cancer survival. Theoretically it is attractive because it is consistent both with late excess mortality and with a cured fraction.

Estimates of parameter values based on follow-up data for 10 years were similar to those obtained using data for the entire follow-up period (Table V). This suggests that the model could be used for prediction of long term results from short term data. However, the relative survival declined during the entire follow-up period (Figure 1) and a cured fraction could not be observed. Hence, extrapolations from the model should be cautiously judged until supported by observed data.

One disadvantage with the lognormal model is that large populations are necessary in order to obtain stable estimates of the parameter values. The 95% confidence interval of the estimated cured proportion for cases aged less than 50 years in the 1961–1963 series, for instance, was found to be 36–47% even though this group consisted of more than 1,900 cases. In order to reduce the standard errors, Boag suggested that the standard deviation of the model might be kept fixed and only the remaining two parameters be estimated. The original 3-parameter model is thus converted into a less flexible 2-parameter model. The rationale for this technique is that the standard deviation might be considered as a constant for a given disease, and was supported by the finding that estimates of the cured proportion were similar even with fairly large variations in the assumed value of the standard deviation (Mould & Boag, 1975). This result was based on studies on carcinoma cervix uteri and on head and neck cancers. However, the estimated value of the standard deviation in the current study (0.71 log-years) was not similar to that estimated in a previous study (0.60 log-years) based on more than 14,000 breast cancer cases from the Norwegian Cancer Registry (Rutqvist *et al.*, 1984). Furthermore, estimates of the cured proportion and the mean of the lognormal distribution were significantly different when the standard deviation was fixed at 0.60 log-years as compared to 0.71 log-years (Table IV). Similar findings were reported by Haybittle (1959). Tests for goodness-of-fit also disclosed significant deviation with the standard deviation fixed at 0.60 log-years (Table III). In view of these inconsistencies the present results support Haybittle's conclusion that the 2-parameter lognormal model is not advisable for analysis of breast cancer survival. The standard deviation is probably affected by the stage distribution and thus probably varies from one case material to another. Therefore, no *a priori* assumption of its value is possible. The reason why some of the previous studies did not produce significant deviations could be that the case materials were smaller or that the studied populations were more homogeneous with regard to disease outcome than is usually the case in breast cancer.

This study confirmed previous information (Rutqvist, 1984) by showing an upward survival trend for breast cancer in Sweden during 1961–1973. The trend was unequally distributed and was only significant for cases aged 50–69 years. The relative survival of patients aged below 50 years and above 70 years showed only minor, insignificant increases. The potential problem of lead time bias should, however, be considered when judging the survival trend. Unfortunately no methods are at present available which permit determination of lead time differences between patient populations diagnosed during different periods.

Lead time bias is not a confounding factor if the outcome of treatment is measured in terms of the proportion of patients who are cured. In the current study, the relative survival declined during the entire follow-up period and a cured group of patients could not be observed. This finding accords with many other long term follow-up studies which have shown that the excess mortality from breast cancer probably persists at least up to 30–40 years after diagnosis (Adair *et al.*, 1974; Baum, 1976; Rutqvist & Wallgren, 1985). The higher relative survival for more recently diagnosed cases (Figure 1) and for young as compared to old cases (Figure 2) might therefore be explained by lead time bias.

To get round this problem, the lognormal model was utilized to interpret the observed survival patterns. According to the model, the survival trend was the result of a 7% increase (95% confidence interval: 0–13%, $P<0.05$) of the cured proportion. The estimated median survival of uncured cases, on the other hand, was similar during 1961–1963 and 1971–1977 (4.5 and 4.6 years). This result suggests that the trend was the result of long-term cures and not merely due to protracted survival with cancer. Similarly the higher relative survival rates for young as compared to old patients were also reflected in higher estimated proportions of cures.

Breast cancer incidence rates have increased in most countries in the Western world during the past decades. It has been suggested that this could be the result of an increased diagnosis of 'biologically benign' breast cancer, i.e. breast lumps exhibiting all histologic characteristics of cancer, but which have relatively benign biological properties (Fox, 1979; Doll & Peto, 1981). An increased proportion of patients with such lesions among the more recently diagnosed cases might explain an increased proportion of 'cured cases' and consequently also an upward survival trend. Due to the limited data on the natural time history of breast cancer, it is not known whether 'biologically benign' breast cancers exist, nor if they have biased the incidence trend. It therefore remains controversial whether the survival trend reported here, is the result of an improved outcome for patients with serious breast cancer or if it is simply artifactual.

### References

ADAIR, F., BERG, J., JOUBERT, L. & ROBBINS, G.F. (1974). Long term follow-up of breast cancer patients. The 30-year report. *Cancer*, **33**, 1145.

BAUM, M. (1976). The curability of breast cancer. *Br. Med. J.*, **1**, 439.

BERKSON, J. & GAGE, R.P. (1952). Survival curve for cancer patients following treatment. *J. Am. Med. Assoc.*, **47**, 501.

BOAG, J.W. (1948). The presentation and analysis of the results of radiotherapy. *Br. J. Radiol.*, **21**, 128 & 189.

BOAG, J.W. (1949). Maximum likelihood estimates of the proportion of patients cured by cancer therapy. *J. Roy. Stat. Soc. (B)*, **11**, 15.

CAMPOS, J.L. (1972). Observations on the mortality from carcinoma of the breast. *Br. J. Radiol.*, **45**, 31.

DOLL, R. & PETO, R. (1981). The causes of cancer: Quantitative estimates of avoidable risks of cancer in the United States today. *J. Natl Cancer Inst.*, **66**, 1191.

EDERER, F., AXTELL, L.M. & CUTLER, S.J. (1961). The relative survival rate. A statistical methodology. *Natl. Cancer Inst. Monogr.*, **6**, 101.

FOX, M.S. (1979). On the diagnosis and treatment of breast cancer. *J. Am. Med. Assoc.*, **241**, 489.

HAYBITTLE, J.L. (1959). The estimation of the proportion of patients cured after treatment for cancer of the breast. *Br. J. Radiol.*, **32**, 725.

HAYBITTLE, J.L. (1965). A two-parameter model for the survival curve of treated cancer patients. *J. Am. Stat. Assoc.*, **309**, 16.

MATTSSON, B. & WALLGREN, A. (1984). Completeness of the Swedish Cancer Register. Non-notified cases recorded on death certificates in 1978. *Acta Radiol. Oncology*, **23**, 305.

MOULD, R.F. & BOAG, J.W. (1975). A test of several parametric statistical models for estimating success rate in the treatment of carcinoma cervix uteri. *Br. J. Cancer*, **32**, 529.

MOULD, R.F., HEARNDEN, T., PALMER, M. & WHITE, G.C. (1976). Distribution of survival times of 12,000 head and neck cancer patients who died with their disease. *Br. J. Cancer*, **34**, 180.

MUELLER, C.B. & JEFFRIES, W. (1975). Cancer of the breast: Its outcome as measured by the rate of dying and causes of death. *Am. J. Surg.*, **182**, 334.

RUTQVIST, L.E. (1984). Increasing incidence and constant mortality rates for breast cancer: Time trends in Stockholm county 1961–1973. *Breast Cancer Res. Treat.*, **4**, 233.

RUTQVIST, L.E. & WALLGREN, A. (1983). Inconsistencies in breast carcinoma registration. An investigation of 855 cases reported to the Swedish Cancer Registry. *Acta Radiol. Oncology*, **22**, 109.

RUTQVIST, L.E. & WALLGREN, A. (1985). Long term survival of 458 young breast cancer patients. *Cancer*, **55**, 658.

RUTQVIST, L.E., WALLGREN, A. & NILSSON, B. (1984). Is breast cancer a curable disease? A study of 14,731 women with breast cancer from the Cancer Registry of Norway. *Cancer*, **53**, 157.